# SPANXA suppresses EMT by inhibiting c-JUN/SNAI2 signaling in lung adenocarcinoma

**DOI:** 10.18632/oncotarget.10088

**Published:** 2016-06-15

**Authors:** Yi-Jing Hsiao, Kang-Yi Su, Yi-Chiung Hsu, Gee-Chen Chang, Jin-Shing Chen, Hsuan-Yu Chen, Qi-Sheng Hong, Shih-Chun Hsu, Po-Hsiang Kang, Chia-Ying Hsu, Bing-Ching Ho, Tsung-Hui Yang, Chia-Yu Wang, Yuh-Shan Jou, Pan-Chyr Yang, Sung-Liang Yu

**Affiliations:** ^1^ Department of Clinical and Laboratory Sciences and Medical Biotechnology, National Taiwan University College of Medicine, Taipei, Taiwan; ^2^ Center of Genomic Medicine, National Taiwan University College of Medicine, Taipei, Taiwan; ^3^ Department of Laboratory Medicine, National Taiwan University Hospital, Taipei, Taiwan; ^4^ Institute of Statistical Science, Academia Sinica, Taipei, Taiwan; ^5^ Faculty of Medicine, School of Medicine, National Yang-Ming University, Taipei, Taiwan; ^6^ Division of Chest Medicine, Department of Internal Medicine, Taichung Veterans General Hospital, Taichung, Taiwan; ^7^ Division of Thoracic Surgery and Department of Surgery, National Taiwan University Hospital and National Taiwan University College of Medicine, Taipei, Taiwan; ^8^ Institute of Biomedical Sciences, Academia Sinica, Taipei, Taiwan; ^9^ Department of Internal Medicine, National Taiwan University Hospital, Taipei, Taiwan; ^10^ Department of Pathology and Graduate Institute of Pathology, National Taiwan University College of Medicine, Taipei, Taiwan; ^11^ Center for Optoelectronic Biomedicine, National Taiwan University College of Medicine, Taipei, Taiwan; ^12^ Institute of Medical Device and Imaging, National Taiwan University College of Medicine, Taipei, Taiwan

**Keywords:** SPANX family, AP-1, SLUG, E-cadherin, metastasis

## Abstract

SPANXA (Sperm Protein Associated with the Nucleus on the X-chromosome, family members A1/A2) acts as a cancer-testis antigen expressed in normal testes, but dysregulated in various tumors. We found that SPANXA is highly expressed in low-invasive CL1-0 cells compared with isogenous high-invasive CL1-5 cells. SPANXA was preferably expressed in tumor tissues and associated with the prolonged survival of lung adenocarcinomas. SPANXA suppressed the invasion and metastasis of lung cancer cells *in vitro* and *in vivo*. By the expression microarray and pathway analysis, we found that the SPANXA-altered genes were enriched in the epithelial–mesenchymal transition (EMT) pathway. SPANXA reduced *SNAI2* expression resulted in up-regulating *E-cadherin*. c-JUN acts as the positive-regulator of EMT. Silencing SPANXA increased c-JUN mRNA expression and blockage of c-JUN led to *SNAI2* down-regulation. Our results clearly characterized SPANXA as an EMT inhibitor by suppressing *c-JUN-SNAI2* axis in lung adenocarcinoma.

## INTRODUCTION

Lung cancer is the leading cause of cancer death worldwide, and adenocarcinoma is the major subtype, accounting for 50% of the incidence of non-small cell lung cancer (NSCLC) [1, 2]. Metastasis is one major hurdle in cancer treatment [3]. During metastasis, cancer cells acquire their invasive capacity by undergoing epithelial–mesenchymal transition (EMT), which enables cells to become motile, and thus, invade adjacent vessels and tissues. The epithelial cells lose cell–cell junctions by down-regulating E-cadherin (CDH1) while acquiring a mesenchymal phenotype, which is characterized by up-regulated mesenchymal proteins, including vimentin (VIM), N-cadherin (CDH2) and fibronectin (FN1). This process is triggered by various signaling pathways controlled by a set of transcription factors, including SNAI1/2, HEY1, TWIST and ZEB1/2 [4–6]. In lung cancer, SNAI2/SLUG is known as a critical EMT inducer that suppresses CDH1 expression and metastasis [7, 8]. In our previous studies, we established a series of lung cancer cell lines CL1-0, CL1-1, CL1-2 and CL1-5, with various malignancies and invasiveness [9]. Using this cell line model and microarray analysis, we identified several invasion-related genes, and characterized their roles in metastasis [7, 10–12]. It is widely now accepted that drug resistance and metastasis of cancer is largely caused by the dysregulation of certain genes [13]. Therefore, we reanalyzed the microarray data of our cell line model and identified SPANXA belonging to cancer-testis antigens (CTAs). The SPANX (Sperm Protein Associated with the Nucleus on the X-chromosome) family encodes highly similar proteins that are exclusively expressed in normal testes as well as in certain tumors [14–17]. In adult human testes, SPANXA/D (A1, A2, B, C, and D) proteins are localized on the nuclear envelope of condensing non-acrosomal regions during spermiogenesis, and expressed in late spermatids and spermatozoa [18, 19]. SPANXA/D proteins have also been found in various tumors, such as melanoma, bladder carcinomas, myeloma, head and neck squamous cell carcinoma (HNSCC), as well as colorectal and prostate cancers [14, 20–23]. However, their roles in spermiogenesis and cancer progression are largely unknown. The nucleotide sequences of the SPANXA/D subfamily generally share similarities of 80%–90% [15, 24]. Moreover, the sequence of *SPANXA1* is identical to that of *SPANXA2*, including introns. Both genes are located at the opposite orientation within the contiguous sequence of Xq27 [17]. Therefore, *SPANXA1* and *SPANXA2* encode the same mRNA and protein, and the biological functions remain to explore.

In this study we analyzed the clinical relevance of SPANXA in lung cancer patients by using the published microarray dataset and Taiwan lung cancer cohort with real-time quantitative reverse transcriptase polymerase chain reaction (qRT-PCR). The effect of SPANXA on metastasis was characterized by *in vitro* and *in vivo* assays. We finally explore the underlying mechanism by which SPANXA regulates the downstream signaling through microarray analysis.

## RESULTS

### SPANXA is upregulated in tumor tissues and associated with prolonged survival in lung adenocarcinoma patients

In our previous studies, we analyzed a series of lung adenocarcinoma cell lines with varying degrees of invasiveness by expression microarrays to identify novel tumor suppressor genes or oncogenes [7, 9, 10, 12]. As with the previous strategy, in a comparison of the expression profiles of low-invasive CL1-0 cells and high-invasive CL1-5 cells, we found a gene, *SPANXA*, which expression was 102 times lower in CL1-5 than in CL1-0 (Supplementary Figure S1). At first, we assessed whether the SPANXA expression is associated with survival of lung adenocarcinoma patients. To address this issue, we conducted a survival analysis using the publicly available microarray dataset, GSE19188. There was a significant correlation between SPANXA expression and overall survival in the dataset of 45 lung adenocarcinoma patients. Patients with low SPANXA expression had a worse overall survival compared with those with high SPANXA expression (log rank test, *p* = 0.03, Supplementary Figure S2A). However, the detection specificity of SPANXA should be carefully considered cause of the high similarity among the SPANX family. Thus we designed the probes of real-time qRT-PCR to detect *SPANXA* and distinguish from other *SPANX* family, especially *SPANXC* that has only seven nucleotides different from *SPANXA*. First, both SPANXA-V5 and SPANXC-V5 expression plasmids were constructed and transfected into HEK293 cells (Supplementary Figure S3A). The customized *SPANXA* and *SPANXC* TaqMan probes as well as SYBR primers detected the corresponding SPANX genes with high specificity, respectively (Supplementary Figure S3B and S3C). Consistent with expression microarray, the differential expression of *SPANXA* in CL1-0 and CL1-5 was further confirmed by qRT-PCR with the TaqMan probe (Supplementary Figure S3D). Next we measured the expression of SPANXC in CL1-0 and CL1-5 cells and found that the SPANXC expression is much lower than SPANXA in both cell lines even if SPANXC is expressed (Supplementary Figure S4). These data indicated that SPANXC does not play an anti-metastatic role at least in CL1-0 and CL1-5 cells. Following experiments, we used SYBR primers to quantify *SPANXA* expression, except for additional notation.

Next, *SPANXA* expression in 97 paired adjacent normal and tumor tissues from the non-small cell lung cancer patients were measured by qRT-PCR with the TaqMan probe (Supplementary Table S1). The result showed that *SPANXA* was dominantly present in tumor tissues (McNemar test, *p* = 0.019, Table 1) and the following subtype stratification found that *SPANXA* expression was upregulated in adenocarcinoma patients (Wilcoxon matched-pairs method, *p* = 0.028, Supplementary Figure S2B).

**Table 1:** SPANXA expression of paired adjacent normal and tumor tissues detected by qRT-PCR

		Normal tissue			*p*-value^#^
		Presence*	Absence*	Total	
Tumor tissue	Presence	5	15	20	0.019
	Absence	4	73	77	
	Total	9	88	97	

* CT of SPANXA greater than 40 cycles was defined as Absence, otherwise as Presence.

# McNemar's test was used for the statistical analyses.

### SPANXA suppresses cell motility and invasiveness *in vitro*

**Figure 1:**
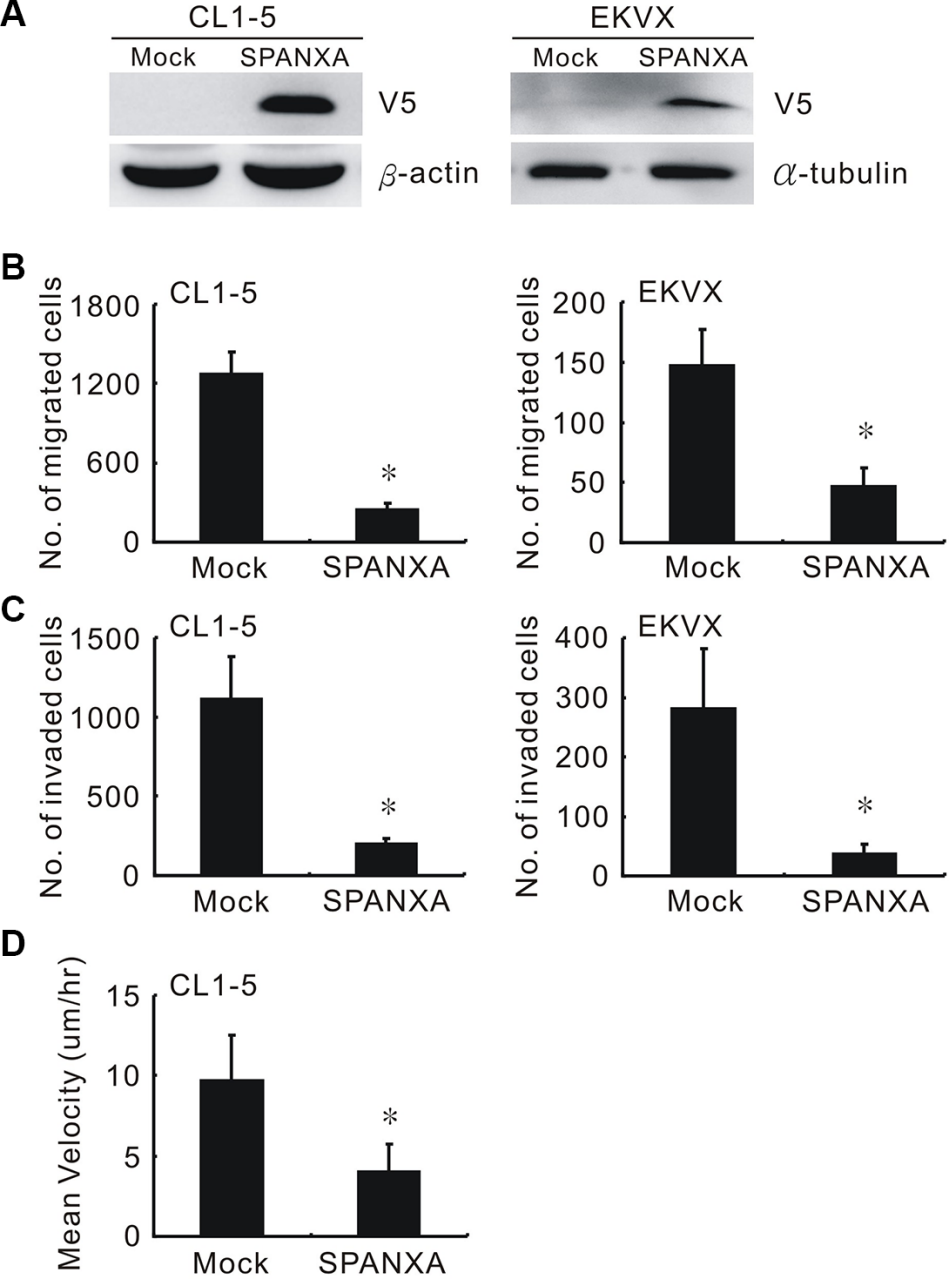
SPANXA suppresses cell migration and invasion (**A**) Establishment of SPANXA-expressing cells in highly invasive cells, CL1-5, and in low SPANXA-expressing cells, EKVX. The SPANXA expression was detected by anti-V5. (**B**) (**C**) Cell migration and invasion abilities of mock or SPANXA-expressing cells measured by Transwell assay. (**D**) Cell motility rate of SPANXA-expressing cells and mock control were measured by single cell tracking. All experiments were performed in triplicates. *P < 0.05 (mean ± SD, *n* = 3).

The role of SPANXA in lung cancer progression has never been fully elucidated. The SPANXA-V5 expression plasmid was transfected into the low SPANXA-expressing CL1-5 and EKVX cells to establish pooled stably SPANXA-expressing cells (Figure 1A and Supplementary Figure S5A). Transwell migration and invasion assays indicated that SPANXA inhibits both migration and invasion abilities in stably SPANXA-expressing cells, CL1-5 and EKVX cells (Figure 1B and 1C) as well as in transient SPANXA-expressing CL1-5 cells (Supplementary Figure S5B) and single clones (Supplementary Figure S5C and S5D). To dissect the underlying cause of SPANXA-mediated invasion suppression, the cell growth evaluation was measured in SPANXA manipulated cell lines. The MTT assay indicated SPANXA does not influence cell proliferation (Supplementary Figure S5E). The single-cell tracking assay revealed that SPANXA suppresses cancer cell motility (Figure 1D). These data clarified that SPANXA inhibits cell migration and invasion.

### Downregulated SPANXA promotes cell migration and invasiveness

**Figure 2:**
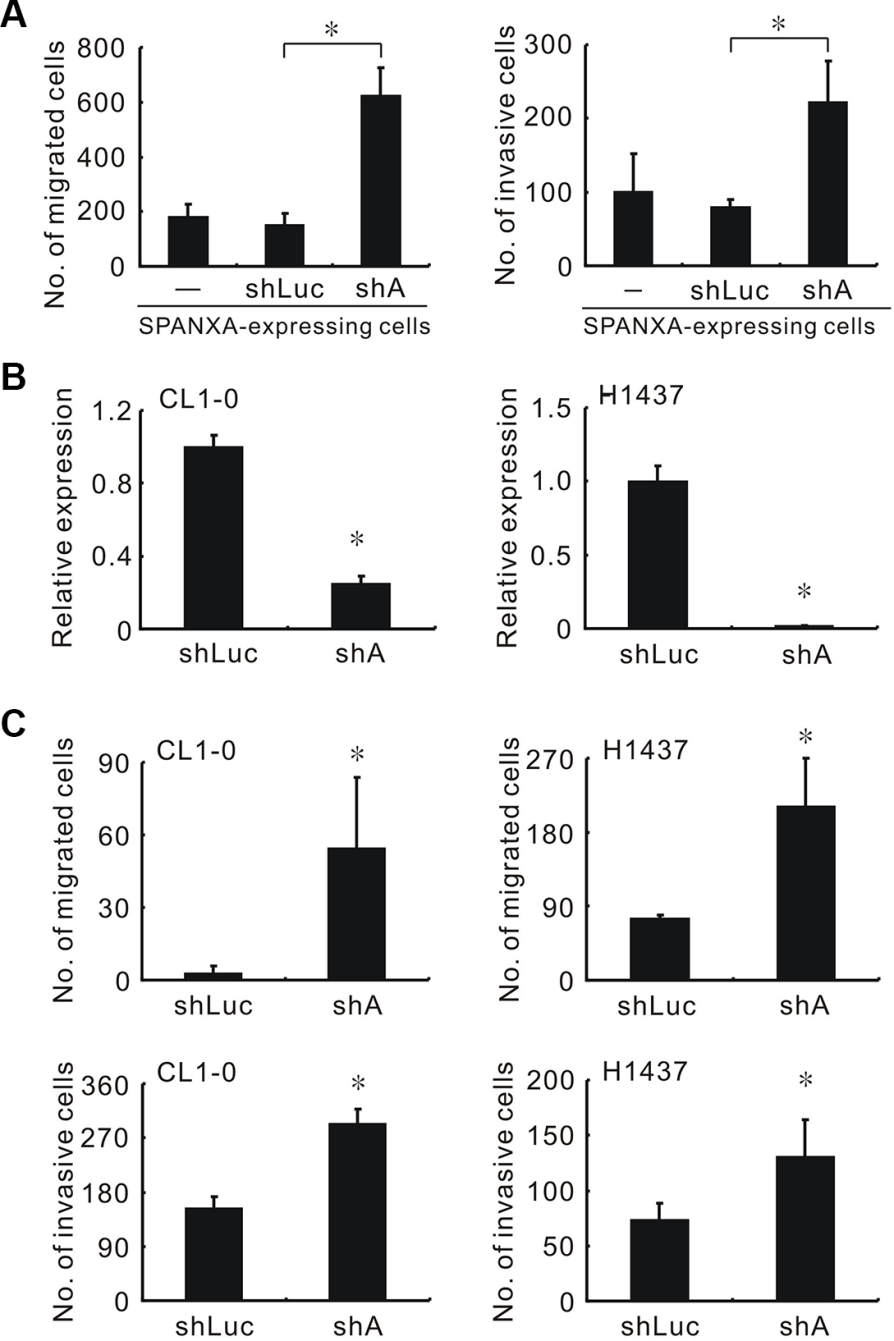
Knockdown of SPANXA increases cell migration and invasion *in vitro* (**A**) Cell migration and invasion abilities of silencing SPANXA in stably SPANXA-expressing CL1-5 cells. (**B**) SPANXA expression in SPANXA-silencing CL1-0 and H1437 cells assessed by qRT-PCR. (**C**) Cell migration and invasion abilities of SPANXA-silencing CL1-0 and H1437 cells. All experiments were performed in triplicates. *P < 0.05 (mean ± SD, *n* = 3).

To evaluate the knockdown efficacy, five shSPANXA lentiviruses which targeted different sites of *SPANXA* were used to infect the SPANXA-expressing HEK293 cells. Only shSPANXA-4 (sh4) reduced the SPANXA expression efficiently (Supplementary Figure S6A). Silencing *SPANXA* enhanced cell migration and invasion in enforced SPANXA-expressing CL1-5 cells (Figure 2A and Supplementary Figure S6B), and in CL1-0 and H1437, which both were highly endogenous SPANXA cells lines (Figure 2B, 2C and Supplementary Figure S5A). In addition to cell migration and invasion, we also investigated whether SPANXA influences tumorigenesis *in vitro*. In anchorage-independent assay, SPANXA-expressing cells formed fewer colonies compared with the mock (Supplementary Figure S6C). These data demonstrated that downregulated SPANXA increases cell migration and invasion in lung adenocarcinoma cells.

### SPANXA inhibits metastasis *in vivo*

**Figure 3:**
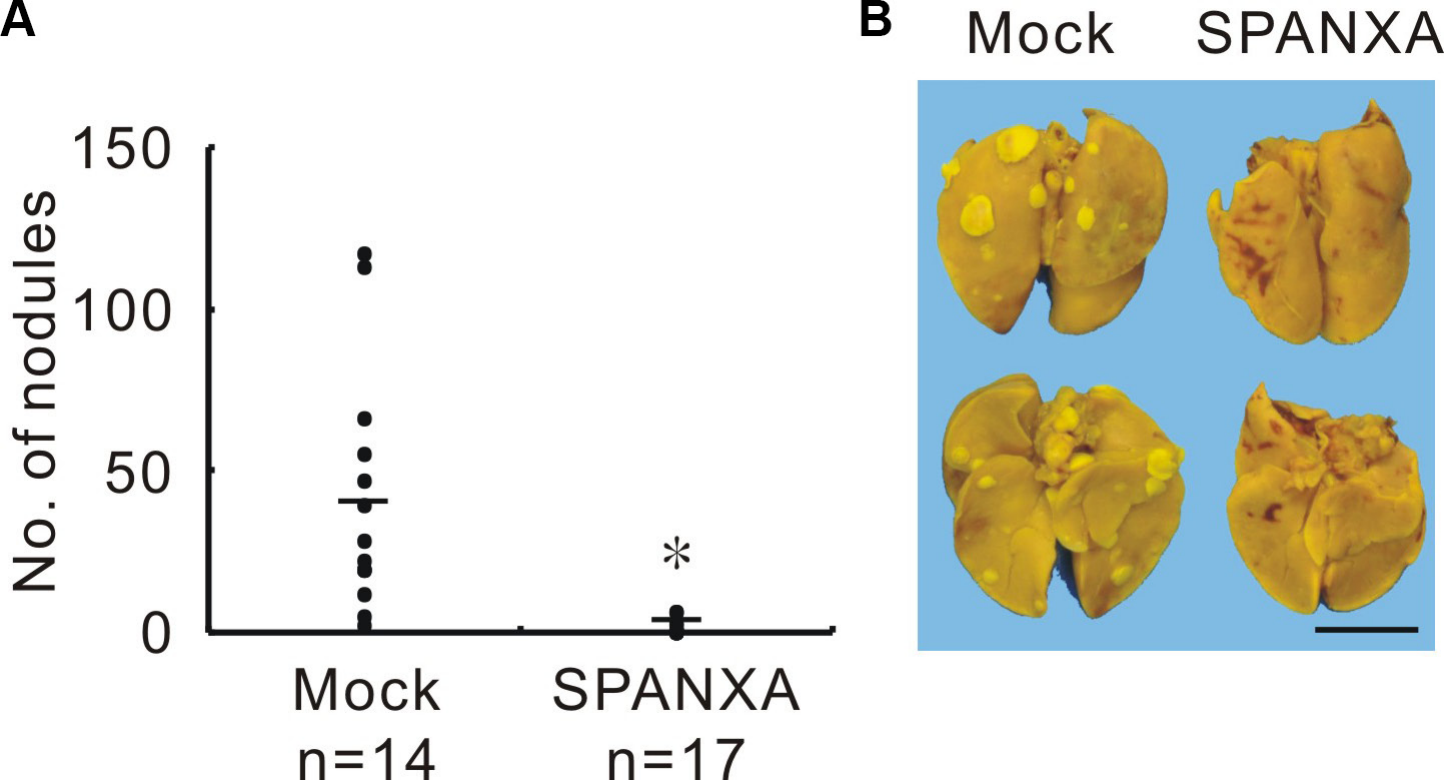
SPANXA attenuates metastasis *in vivo* (**A**) *In vivo* tumor metastasis effect of SPANXA was analyzed by an experimental metastasis assay with stably SPANXA-expressing CL1-5 cells and mock control cells, which were intravenously injected into NOD-SCID mice. The metastatic tumor nodules were calculated. *P < 0.05. (**B**) Image of lung surface nodules. Anterior lungs showed on the upper part and posterior lungs on the lower part. Scale bar, 0.5 μm.

To evaluate the role of SPANXA *in vivo*, we injected the stably SPANXA-expressing or mock control cells into the lateral tail vein of NOD-SCID mice. The mice were sacrificed after five weeks, and the lungs were examined for metastasis formation. Compared with mock mice, the nodules decreased significantly in SPANXA mice (Figure 3A and 3B). The representative lungs were stained with hematoxylin and eosin (H&E) for histological analysis (Supplementary Figure S7). These results revealed that SPANXA plays a role in metastasis suppression *in vivo*.

### SPANXA is mainly involved in EMT pathway

**Figure 4:**
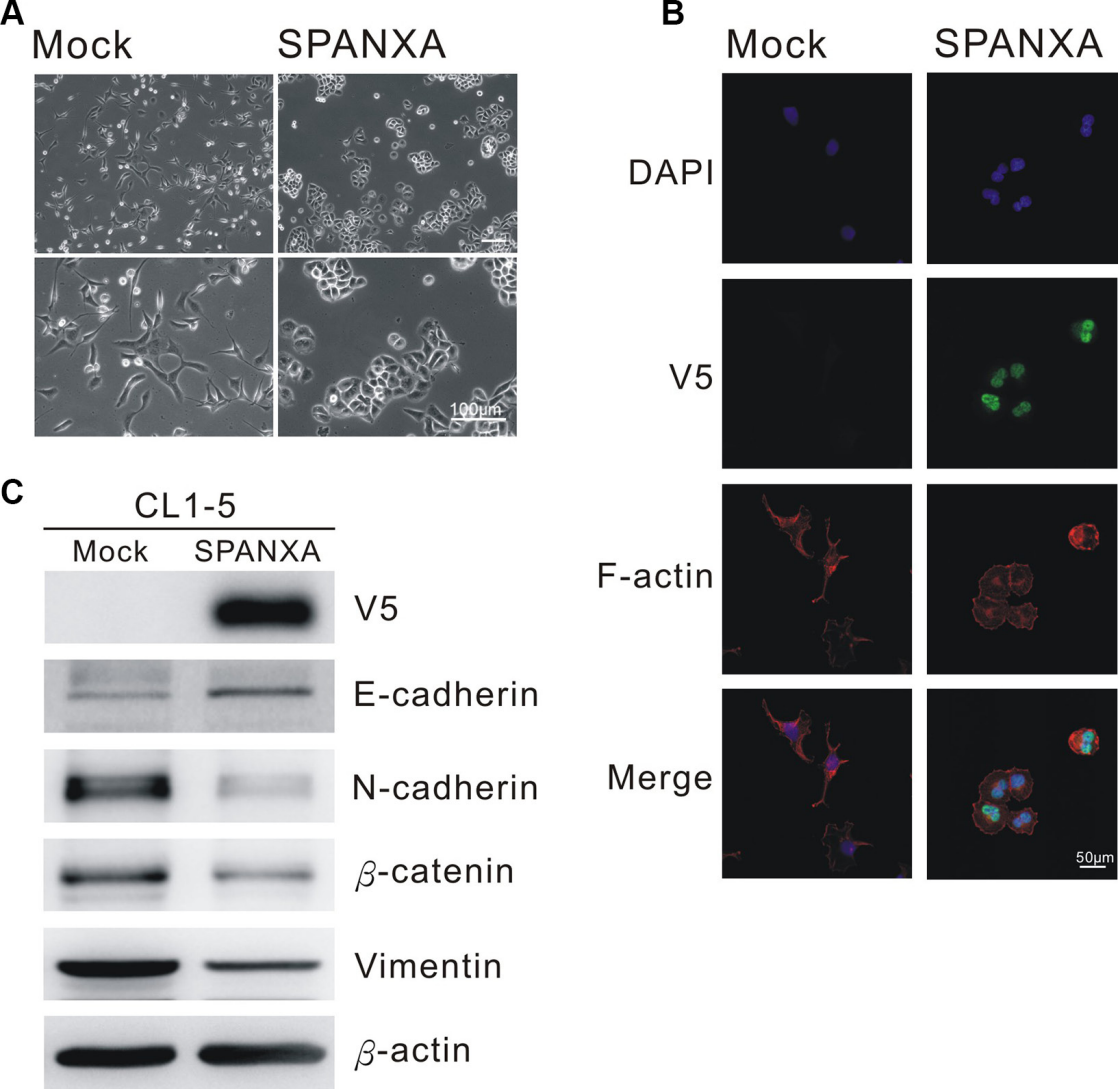
SPANXA inhibits EMT process (**A**) Cell morphology of stably SPANXA-expressing CL1-5 cells and mock control cells. (**B**) Anti-V5 and anti-F-actin immunofluorescence staining of mock control and stably SPANXA-expressing CL1-5 cells. (**C**) Expression of EMT marker in stably SPANXA-expressing CL1-5 cells was assessed by immunoblot assays. All experiments were performed in duplicates.

To dissect the underlying mechanism through which SPANXA suppresses metastasis, we performed oligonucleotide expression microarrays to profile differentially expressed genes in stably SPANXA-expressing cells. There were 1024 genes with a 2-fold change (*p* < 0.05) applying to MetaCore enrichment pathway analysis software, and the top 10 most affected pathways were identified (Supplementary Table S2). Surprisingly, the altered genes were largely enriched to EMT pathway: four pathways belonged to EMT pathways and five were EMT-related pathways. Following verification, the cell morphology of stably SPANXA-expressing cells changed markedly into the epithelial type from the original CL1-5 mesenchymal-like type (Figure 4A). The F-actin remodeling occurred in the SPANXA-expressing cells, which evidently had less filapodia compared with the mock control (Figure 4B). The anti-V5 immunoflorescence staining results indicated that SPANXA localizes mostly in the nucleus (Figure 4B). When SPANXA was overexpressed in CL1-5 cells, the epithelial marker, E-cadherin was upregulated, whereas other mesenchymal proteins were downregulated (Figure 4C). Otherwise, knockdown of *SPANXA* expression reduced the protein and mRNA expression of E-cadherin, and increased mesenchymal markers N-cadherin, β-catenin and Vimentin in CL1-0 cells (Supplementary Figure S8A and S8B). Overall, these data clearly demonstrated that SPANXA negatively regulates EMT in lung cancer cells.

### SPANXA inhibits cell invasion by reducing SNAI2/SLUG expression

**Figure 5:**
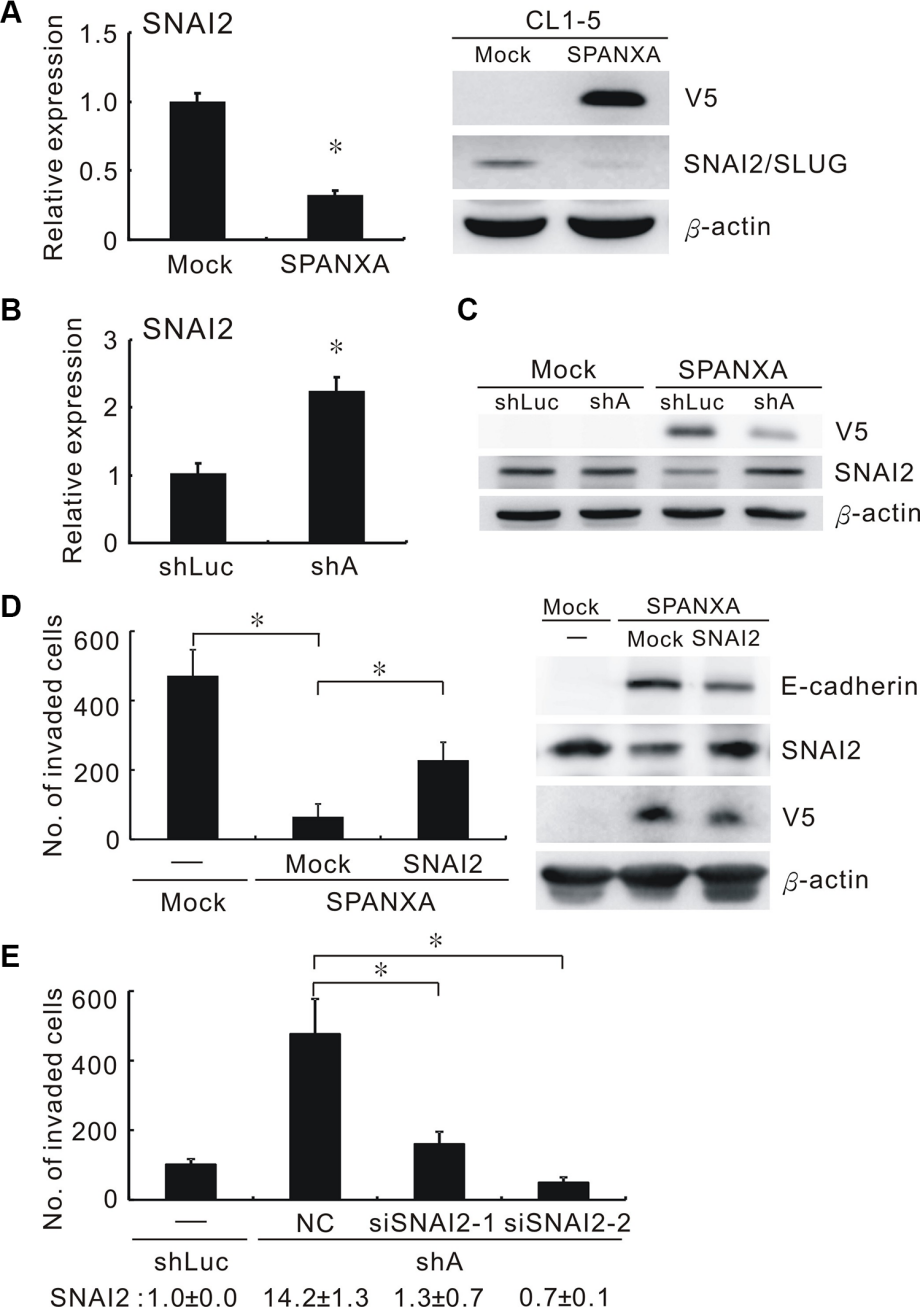
SPANXA represses cell invasion through deregulating SNAI2 (**A**) Protein and mRNA expressions of SNAI2 in stably SPANXA-expressing CL1-5 cells and mock control cells assessed by qRT-PCR and Western blot assays. *P < 0.05 (mean ± SD, *n* = 3). (**B**) RNA expression of SNAI2 in SPANXA-silencing CL1-0 cells and shLuc control cells assayed by qRT-PCR. *P < 0.05 (mean ± SD, *n* = 3). (**C**) The stably SPANXA-expressing CL1-5 cells were infected with shSPANXA lentivirus and assayed for SNAI2 protein expression by Western blot assays. The experiment was performed in duplicates. (**D**) Cell invasion and expression of SNAI2 and E-cadherin of stably SPANXA-expressing CL1-5 cells was assessed in presence of SNAI2 overexpression. *P < 0.05 (mean ± SD, *n* = 3) (**E**) Cell invasion of stably SPANXA-silencing CL1-0 cells was assessed under SNAI2 silencing. The lower row indicated *SNAI2* RNA expression assayed qRT-PCR. *P < 0.05 (mean ± SD, *n* = 3). All assays were performed in two independent experiments.

Through microarray analysis, we identified several genes involved in the EMT-related pathways; one was *SNAI2*, which was downregulated to 0.22-fold in SPANXA-expressing cells (Supplementary Table S3). The transcriptional factor SNAI2 was a critical EMT inducer that suppresses its target, E-cadherin. Both mRNA and protein expressions of *SNAI2* were obviously reduced in the stably SPANXA-expressing cells compared with the mock control cells (Figure 5A). As expected, E-cadherin was also downregulated detected by microarray and qRT-PCR assays (Supplementary Table S3). By contrast, silencing SPANXA in CL1-0 cells enhanced *SNAI2* expression and reduced E-cadherin expression (Figure 5B and Supplementary Figure S8B). Moreover, SPANXA knockdown in stably SPANXA-expressing cells rescued *SNAI2* downregulation (Figure 5C). To clarify the role of SNAI2 in SPANXA-mediated invasion suppression, we ectopically expressed SNAI2 in stably SPANXA-expressing cells and assessed cell invasion. The invasive ability suppressed by SPANXA was partly regained by restoring SNAI2 (Figure 5D). By contrast, when we used siRNA to suppress SNAI2 expression, which was upregulated in stably SPANXA-silencing CL1-0 cells, the invasive ability was inhibited (Figure 5E). These results revealed that SPANXA inhibits cell invasion by transcriptionally reducing SNAI2 expression.

### SPANXA suppresses c-JUN expression leading to SNAI2 downregulation

**Figure 6:**
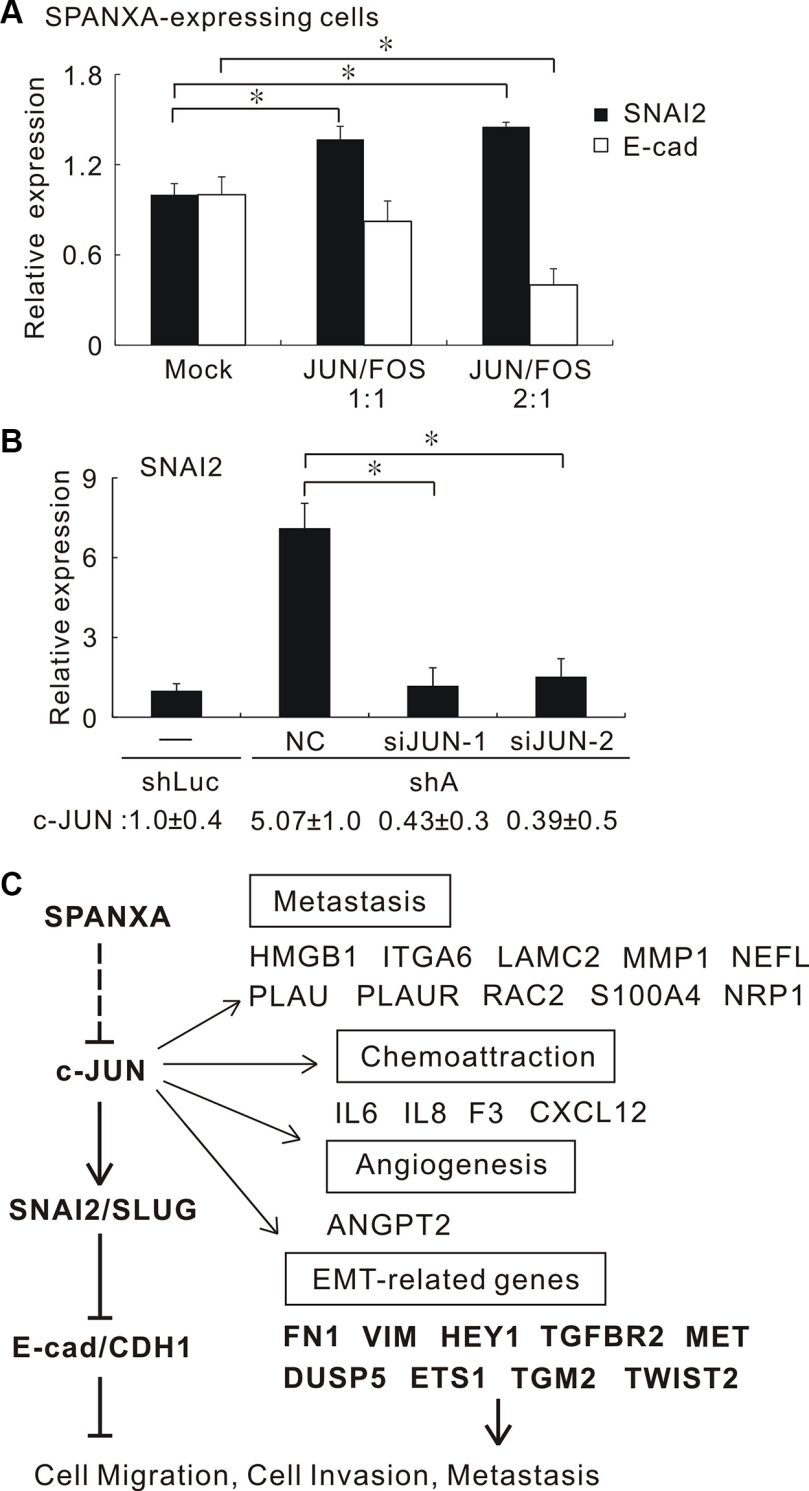
SPANXA suppresses SNAI2 expression and EMT through repressing c-JUN (**A**) The different ratios of c-JUN and c-FOS were ectopically introduced into stably SPANXA-expressing CL1-5 cells. The *SNAI2* and *E-cadherin* expressions were measured by qRT-PCR. *P < 0.05 (mean ± SD, *n* = 3). (**B**) Knockdown of c-JUN in stably SPANXA-silencing CL1-0 cells reduced the *SNAI2* expression. The lower row indicated the *c-JUN* expression. *P < 0.05 (mean ± SD, *n* = 3). (**C**) Model of SPANXA-regulating network. SPANXA inhibits the c-JUN/SNAI2/CDH1 signalling and EMT process. The dotted line indicates the unexplored part of SPANXA regulation. All assays were performed in two independent experiments.

Previous studies reported that the β-catenin/TCF is responsible for transcription of SNAI2 [25, 26]. Although SPANXA reduced β-catenin expression, silencing β-catenin did not significantly decrease the SNAI2 expression in SPANXA-silencing CL1- 0 cells (Supplementary Figure S9). To identify which transcriptional factor responsible for *SNAI2* suppression, we analyzed 1024 SPANXA-altered genes from the microarray to build the SPANXA-regulating network by transcriptional regulation analysis in MetaCore. The SNAI2-involved network was sorted out. We found c-JUN to be the key mediator in this network, and these genes in the network are enriched in cell motility (*p* = 1.20e–22) (Supplementary Table S4). Generally, c-JUN dimerized with c-FOS to form AP-1. We first assessed c-JUN and c-FOS expressions in the stably cell lines. The data showed that SPANXA regulates c-JUN expression but not the c-FOS in both SPANXA-expressing and SPANXA-silencing cells (Supplementary Figure S10A and S10B). Additionally, the c-JUN mRNA is up-regulated in SPANXA-expressing cells and down-regulated in SPANXA-silencing cells (Supplementary Figure S10C). The AP-1 reporter assay showed that SPANXA significantly decreases the AP-1 activity (Supplementary Figure S10D). These data suggested that SPANXA attenuates AP-1 activity mainly through c-JUN suppression. We delivered a different ratio of c-JUN and c-FOS expression plasmids into stably SPANXA-expressing CL1-5 cells. Re-expressed AP-1 increased *SNAI2* expression and reduced E-cadherin expression (Figure 6A and Supplementary Figure S11). Moreover, silencing c-JUN repressed *SNAI2* upregulation in SPANXA-silencing CL1-0 cells (Figure 6B). Next we identified the AP-1 binding motif (TGACTCA) in the promoter of human SNAI2 (NCBI accession No. NT_008183.20). The chromatin immunoprecipitation result showed that the region of AP-1 binding site is occupancy of c-JUN (Supplementary Figure S12). These results indicated that SPANXA inhibited *SNAI2* expression via suppressing c-JUN at transcriptional level.

We evaluate the impact of SPANXA on the AP-1 transcriptional landscape by expression microarray and pathway analysis. There are 397 genes which are significantly down-regulated with 2-fold change in SPANXA-expressing cells; 583 genes are considered as AP-1/c-JUN regulating targets annotated by MetaCore software (GeneGo, St. Joseph, MI). The intersection of these AP-1/c-JUN targets and the SPANXA down-regulated genes are 31 genes. The SPANXA down- regulated genes are significantly enriched in AP- 1/c-JUN regulating targets (Chi-square test, *p* = 1.50 × 10^−09^, total gene number = 21, 123) (Supplementary Table S5). Thirty-one AP-1/c-Jun targets are down-regulated in stably SPANXA-expressing cells listed (Supplementary Table S3). This data indicated that AP-1 alteration is a major function of SPANXA.

Figure 6C showed the signaling network of SPANXA-mediated tumor suppression in which SPANXA downregulates the c-JUN expression and a set of AP-1 targets associated with EMT, angiogenesis, chemoattraction and metastasis. The expression of EMT-related genes was confirmed by qRT-PCR (Supplementary Table S3). These results revealed that SPANXA suppresses *SNAI2* and reverses EMT through *c-JUN* suppression.

## DISCUSSION

Cancer-testis antigens (CTAs) are a potential target for developing the anti-tumor vaccines by their testis/tumor-biased expression pattern. Recently, emerged reports implied that certain CTAs are directly involved in tumorigenesis, metastasis and prognosis [27–29]. SPANX was first discovered as CTAs, which are widely distributed in tumors, such as in lung, liver, and colon cancers, as well as melanoma, but not in normal tissues, except for the testis [14, 16, 30]. The SPANX expression might be positively associated with metastasis in colorectal cancer and in metastatic melanoma [17, 31]. A research indicated that SPANX was seemingly involved in cancer stem-like cells and malignant progression [32]. The members of SPANX family share a high similarity that restricts functional investigation for SPANXA. Herein, we characterize the biological role and clinical significance of SPANXA, and demonstrate SPANXA blocks EMT and leads to invasive suppression through suppressing AP1 signaling.

At first, we attempted to distinguish SPANXA from its family members. Beyond the limitation of a high degree of similarity between SPANXA and SPANXC, we tested the commercially available antibodies for SPANXA. However, they failed to detect the ectopic expression of SPANXA-V5. Salemi *et al.* underscored this difficulty in differentiating SPANX subfamilies by antibodies because of the high homology among SPANX family members [23]. Hence, we designed the SPANXA-specific qRT-PCR probe/primer sets. We found that SPANXA was associated with prolonged overall survival of the patients with lung adenocarcinoma, and it was predominantly presented in lung tumor parts. This unique tumor/normal expression pattern of SPANXA is quite different from other tumor suppressors which are often down-regulated in tumors. The reason for SPANXA dysregulated in lung adenocarcinoma is still unclear. But our data clearly indicated that SPANXA is a metastasis suppressor in lung adenocarcinoma. To our best knowledge, this is the first study to characterize the functional role of SPANXA in cancers.

SPANXA is an acidic protein and associates with other nuclear envelope proteins, but shows no significant similarity with known nuclear proteins or nucleic acid-binding proteins in the spermatid [16, 19]. These studies implied that SPANXA may interact with nucleic acids or other proteins in performing its functions. Indeed, our findings demonstrated that ectopic SPANXA is largely located at nucleus and suppresses SNAI2 at transcriptional level. And c-JUN is responsible for *SNAI2* expression and EMT suppression. c-JUN is a basic region-leucine zipper protein belonging to one member of the AP-1 family. Transcription factor AP-1 is a dimer of c-JUN and c-FOS families [33]. The induction of c-JUN expression and activity leads to tumor progression and development [34]. There are several AP-1 binding sites on SNAI2 promoter and SNAI2 is a direct transcriptional target of c-JUN in murine [35, 36]. In breast cancer, SNAI2 was expressed in an AP-1-dependent manner [37]. Furthermore, c-JUN expression was found to parallel with SNAI2 expression, and correlated with a mesenchymal gene signature in a panel of melanoma cell lines and a patient cohort [38]. In this study we identified the AP-1 motif in the human promoter of SNIA2 occupancy of c-Jun and proved that the SPANXA-mediated EMT suppression is AP1-dependent. These evidences suggest that c-JUN/SNAI2 signaling is a major pathway contributed to SPANXA-mediated invasive suppression.

The SPANXA-inhibiting c-JUN targets are involved in metastasis, chemoattraction, angiogenesis and EMT (Figure 6C). Liu *et al.* reported that c-JUN induces the EMT process and directly activates a group of genes including *TWIST2, TGM2, DUSP5* and *ETS1* [39]. In agreement with this report, these four genes are down-regulated after ectopic SPANXA expression. In addition, several protumoral targets of AP-1/c-JUN were identified, implying that SPANXA suppresses tumorigenesis. For example, angiopoietin-2 (ANGPT2) is a proangiogenic protein facilitating neoangiogenesis [40]. ANGPT2 promoted metastasis of breast cancer through SNAI1 induction and E-cadherin inhibition [41]. High-mobility group protein B1 (HMGB1) is a chromosomal protein promoting tumor progression. HMGB1 is positively associated with the pathological grade and metastasis of liver cancer, and correlated with lung cancer patients’ survival [42, 43]. The SPANXA-regulating network inhibits these protumoral genes that points out the importance of SPANXA. In fact, there are certain drawbacks in our study. Only one functional SPANXA shRNA may have an unavoidable off-target effect in the SPANXA-silencing experiments. Another concern was that we could not exclude the compensative effect attributed to other similar members. However, SPANXA obviously suppressed EMT process, inhibited SNAI2 and c-JUN expressions in our model cell lines. The mechanism regarding how SPANXA suppresses c-JUN warrants further investigation. In conclusion, we provided evidence on and insight into the functional anti-metastasis roles of SPANXA, and demonstrated that SPANXA inhibits c-JUN/SNAI2 signaling resulting in EMT reversal. These results suggest that SPANXA may be a potential target for therapeutic strategies targeting metastasis of lung cancer.

## MATERIALS AND METHODS

### Clinical specimens

Ninety-seven paired of lung cancer and adjacent normal lung tissues obtained from Taichung Veterans General Hospital and National Taiwan University Hospital were used to measure the *SPANXA* expression. All lung cancer patients were staged in accordance with the American Joint Committee on Cancer Staging (AJCC) and the histology was performed with World Health Organization standards. This study was approved by the institutional review board of Taichung Veterans General Hospital and National Taiwan University Hospital.

### Cell culture

Human lung adenocarcinoma invasion model cell lines, CL1-0 and CL1-5, were established as described in our previous study [9]. Lung adenocarcinoma cell lines EKVX and H1437 were cultured in Dulbecco's Modified Eagle Medium (Life technologies, Carlsbad, CA) with 10% fetal bovine serum (Life technologies, Carlsbad, CA) and 1% penicillin and streptomycin (100 mg/mL). All cell lines were incubated at 37°C in a humidified 20% O_2_/5% CO_2_ environment.

### Plasmid construction and transfection

The SPANXA (GeneBank NM_013453) coding region was amplified and cloned into pEF6/V5-His-TOPO vectors (Life technologies, Carlsbad, CA). The sequence of SNAI2, c-JUN and c-FOS were cloned into the pcDNA3.1/V5-His-TOPO vectors. The cells were transfected with plasmids or siRNA by using Lipofectamine^TM^ 2000 or RNAiMAX (Life technologies, Carlsbad, CA), in accordance with the manufacturer instructions. For transient transfection, the transfected cells were harvested 48 h later; for stably SPANXA-expressing transfectants, the cells were selected with 9 mg/mL Blasticidin S; for stably SPANXA-repressing transfectants, cells were treated with 2.5 μg/mL puromycin (Life technologies, Carlsbad, CA).

### Lentiviral-based shRNA and siRNA

The plasmids of shRNA for SPANXA were supplied by National RNAi Core Facility Platform (Academia Sinica, Taipei, Taiwan). They provided only 5 sequences to users. The clone ID of the fourth shRNA (sh4) for SPANXA was TRCN0000203935, and the target sequence was 5′-CGCTACAGGAGGAACTTTAAA-3′. The CL1-0 and H1437 cells were seeded onto 2 × 10^4^ plates for 24 h before infection. Viral infection was performed according to the manufacturer protocol. SNAI2 and c-JUN siRNA were purchased from Invitrogen (Life technologies, Carlsbad, CA).

### Western blot analysis

The quantitated cell lysates were separated on 8%–12% SDS polyacrylamide gels and transferred onto PVDF membranes. After blocking for 1 h, the membranes were incubated sequentially with anti-V5 antibody (Life technologies, Carlsbad, CA), anti-β-actin antibody (Abcam, Cambridge, MA), anti-CDH1 antibody (BD Biosciences, San Diego, CA), anti-VIM antibody, anti-β-catenin ( Millipore, Temecula, CA), anti-CDH2 antibody (BD Bioscience, San Diego, CA), anti-SNAI2 antibody, anti-c-JUN antibody (Santa Cruz Biotechnology, Dallas, TX), and anti-β-tubulin antibody (Abcam, Cambridge, MA) in PBST. After washing for 3 times, the bound antibody was detected using the Enhanced Chemiluminescence System.

### *In vitro* migration and matrigel invasion assay

The migration and invasion abilities were determined using a Transwell apparatus without or with Matrigel-coated membranes (BD Biosciences, Franklin Lakes, NJ). The migration and invasion assay procedure was detailed in a previous study [11]. The total number of attached cells was counted. All experiments were performed in triplicate. Single cell migration assay was performed as following description. Mock and stably SPANXA-expressing CL1-5 cells were seeded 5000 cells/per well in 6-well culture plate for overnight attachment. Wash wells with PBS twice and replace fresh medium. Set up the focus and sites for 25 position /per well, and take the picture every 1 hour in 24 hours. The high content screening software MetaMorph (Molecular Devices, Sunnyvale, CA) was used to analyze every single cell tracking and mean velocity.

### qRT-PCR

The expression level of SPANXA was detected by qRT-PCR on ABI prism 7900 sequence detection system (Applied Biosystems, Branchburg, NJ), performed in accordance with the manufacturer instructions. For the SYBR Green methods, the SPANXA primers used were the following: forward primer SPANXA-F: 5′-AACGAGGCCAACGAGATGAT-3′ and reverse primer SPANXA-R: 5′-CTAGTATGGTCGAGGACTCAGAT GTT-3′ as well as the TATA box-binding protein (TBP) TBP-F: 5′-CACGAACCACGGCACTGATT-3′ and TBP-R: 5′-TTTT CTTGCTGCCAGTCTGGAC-3′. TBP was used as the internal control. For TaqMan methods, the sequences of customized SPANXA and SPANXC detection probes were as follows: SPANXA forward primer: 5′-CGGGTCTGAGTCCCCAGTT-3′, reverse primer: 5′- TC CCCTGTGATTCCAACGA-3′, and the reporter sequence: 5′-CGGCATCATCTCGTTGGC-3′; SPANXC forward primer: 5′-CGGGTCTGAGTCCCCAGTT-3′, reverse primer: 5′-TCCCCTGTGATTCCAACGA-3′, and the reporter sequence: 5′-CGGCATCGTCTCATTCAC-3′. The TBP detection probe (Assay ID: Hs00427621_m1) was used as the internal control, which was supplied by the ABI company (Applied Biosystems, Branchburg, NJ). The relative expression level of interest, compared with that of TBP, was defined as – ΔCT_Interest_ = – (CT_Interest_ –CT_TBP_), whereas the relative expression ratio of interest between different treatments was calculated as 2^− ΔΔCT^. The primers of the EMT-related genes are listed in Supplementary Table S6.

### Microarray analysis

For the microarray experiments, the total RNA of stably SPANXA-expressing CL1-5 cells and mock control was extracted and subjected to expression microarray analysis by using a Human Genome U133 Plus 2.0 GeneChip in accordance with the manufacturer instructions (Affymetrix, Santa Clara, CA). Experimental variations resulting from differences between microarrays were reduced by scaling the intensity values of the probes in each microarray by employing a quantile-normalization method. Raw data were normalized by GC-RMA and subjected to the unpaired Student *t* test (*P* < 0.05) through GeneSpring GX 12.6 (Silicon Genetics, Redwood City, CA). The 1024 genes (2-fold change) that were expressed significantly between groups were subjected to pathway analysis by using MetaCore (version 6.16, GeneGo, St. Joseph, MI). These array data were uploaded onto GEO (GSE72323).

### Metastasis in SCID mice

For *in vivo* metastasis assay, 6-week-old severe combined immunodeficiency (SCID) mice (supplied by the animal center at the College of Medicine, National Taiwan University, Taipei, Taiwan) were housed (6 mice per cage) and fed ad libitum with autoclaved food. All of the experiments were approved by the Board of Animal Welfare of the National Taiwan University College of Medicine. The stably SPANXA-expressing or mock cells were resuspended at 9 × 10^5^ cells/100 μL PBS and injected into the lateral tail vein with a single-cell suspension in PBS. At 5 weeks post-injection, the mice were sacrificed. Their lungs were harvested, fixed in Bouin's solution, and photographed. The numbers of metastatic tumor nodules in the lungs was counted under a dissecting microscope. The representative lung tumors were removed, fixed, and embedded in paraffin. A 5 μm section was stained with H&E for histological analysis.

### Immunofluorescence

Stable SPANXA-expressing CL1-5 cells were seeded onto chamber slides (1 × 10^3^ cells/well) before they were incubated for 48 h. After formalin fixing, the cells were blocked and incubated with anti-V5 at 4°C for 16 h. They were then washed using a blocking buffer. Finally, the cells were detected after incubation by using the secondary antibody Alexa Fluor® 488 (Life technologies, Carlsbad, CA) for 16 h at 4°C. The cell nucleus was counterstained with DAPI, and F-actin was counterstained with Phalloidin (Life technologies, Carlsbad, CA). The cells were imaged on Nikon confocal C1 system.

### Statistical analysis

The statistical tests without annotations were two-sided Student *t* test, and *P*-value < 0.05 was considered statistically significant. When appropriate, the results are presented as the means ± SD. SPSS version 13.0 was used for above statistical analyses. Early-stage lung adenocarcinoma cohort (45 patients) used for validation was from GEO database (GSE19188) [44]. We focused the 45 adenocarcinoma lung cancer in the cohort. A patient's risk score was calculated as the sum of the levels of expression of 220922_s_at (SPANXA1/A2, SPANXB1/B2, SPANXC and SPANXF1) and 224032_x_at (SPANXA1/A2 and SPANXC). Patients were classified as having a high-risk gene signature or a low-risk gene signature, with the median of risk score as the threshold value. Survival curves for both groups were obtained by the Kaplan-Meier method and were compared using the log-rank test. Both the log-rank test was two-sided, and a *P*-value *<* 0.05 was considered statistically significant.

## SUPPLEMENTARY MATERIAL FIGURES AND TABLES


