# Supplementary material for: SPANXA suppresses EMT by inhibiting c-JUN/SNAI2 signaling in lung adenocarcinoma
Source: Oncotarget. 2016 Jun 15;7(28):44417–29. doi: 10.18632/oncotarget.10088 (PMC5190107; doi:10.18632/oncotarget.10088)
Supplement: Supplementary file 1 [file oncotarget-07-44417-s001.pdf]

SPANXA suppresses EMT by inhibiting c-JUN/SNAI2 signaling in lung adenocarcinoma

Supplementary Materials

SUPPLEMENTARY MATERIALS AND METHODS

Cell proliferation

SPANXA-expressing and mock CL1-5 cells were seeded onto 96-well plates (1000 cells/well). After culturing for different durations, cell proliferation was evaluated by thiazolyl bluetetrazolium bromide (MTT) assay according to the manufacturer`s protocol (Chemicon). Briefly, 10 ul of the MTT solution (5mg/mL) was added to each well, the cells were cultured for another 4 hours at 37ºC. Then 100 ul of 0.04N HCl in isopropanol was added to each well and mixed vigorously to solubilize colored crystals. The absorbance at 570 nm (630 nm as the reference) was measured by using a multi-well scanning spectrophotometer Victor3 (Perkin-Elmer, Boston, MA). Experiments were performed three times in triplicate.

Anchorage-independent colony formation

SPANXA-expressing and mock CL1-5 cells were seeded (1000 cells/well) in 3 ml 0.35% L.M.P. agarose (Life technologies, Carlsbad, CA) onto 6-well plates which is coated with 2 ml 0.7% L.M.P. agarose. The plates were incubated for 1 month and then stained with 0.05% crystal violet. The diameter of colonies greater than 5 pixels were counted under digital camera images.

AP-1 reporter assay

3X AP-1 pGL3 construct (three canonical AP-1 binding sites: TGACTGA) was obtained from Addgene (Vasanwala FH et al., 2002). All transfections were carried out in triplicate in six-well plates. Stably SPANXA-expressing cells were seeded 24 hr before transfection. The luciferase reporter construct were cotransfected with the Renilla control plasmid (pRL-TK; Promega, Madison, WI) into SPANXA-expressing or mock CL1-5 cells by Lipofectamine 2000 (Life technologies, Carlsbad, CA). After 48 hr incubation, the Dual-Glo luciferase substrate (Promega, Madison, WI) was added to each well and the luminescent signals were measured by multi-well scanning spectrophotometer Victor3 (Perkin-Elmer, Boston, MA) according to the manufacturer’s instructions. The activity of Renilla luciferase was used as an internal control to normalize transfection efficiency.

Chromatin immunoprecipitation

Chromatin immunoprecipitation was performed according to the manufacturer’s instructions (Millipore, Temecula, CA). The primers for the AP-1 motif are 5′-GCTTGCGTTTTTACCACATGGT-3′ and 5′-GTAACACGTATGCCCGTGTGA-3′. The antibody using as positive control is the rabbit polyclone anti-RNA polymerase II which is contained in the kit. Anti-c-Jun is a mouse monoclone (Abcam, Cambridge, MA). All experiments were performed in triplicate by Real-time PCR.

REFERENCE

1. Vasanwala FH, Kusam S, Toney LM, Dent AL. Repression of AP-1 function: a mechanism for the regulation of Blimp-1 expression and B lymphocyte differentiation by the B cell lymphoma-6 protooncogene. J Immunol. 2002; 169:1922–1929.

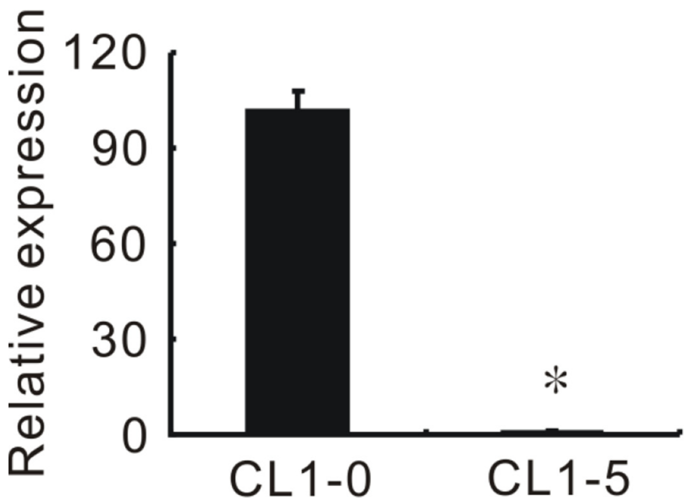

Supplementary Figure S1: SPANXA is highly expressed in low invasive lung cancer cells. SPANXA expression in CL1-0 and CL1-5 cells was measured by expression microarrays. *$P < 0.05$ (mean ± SD, $n = 3$).

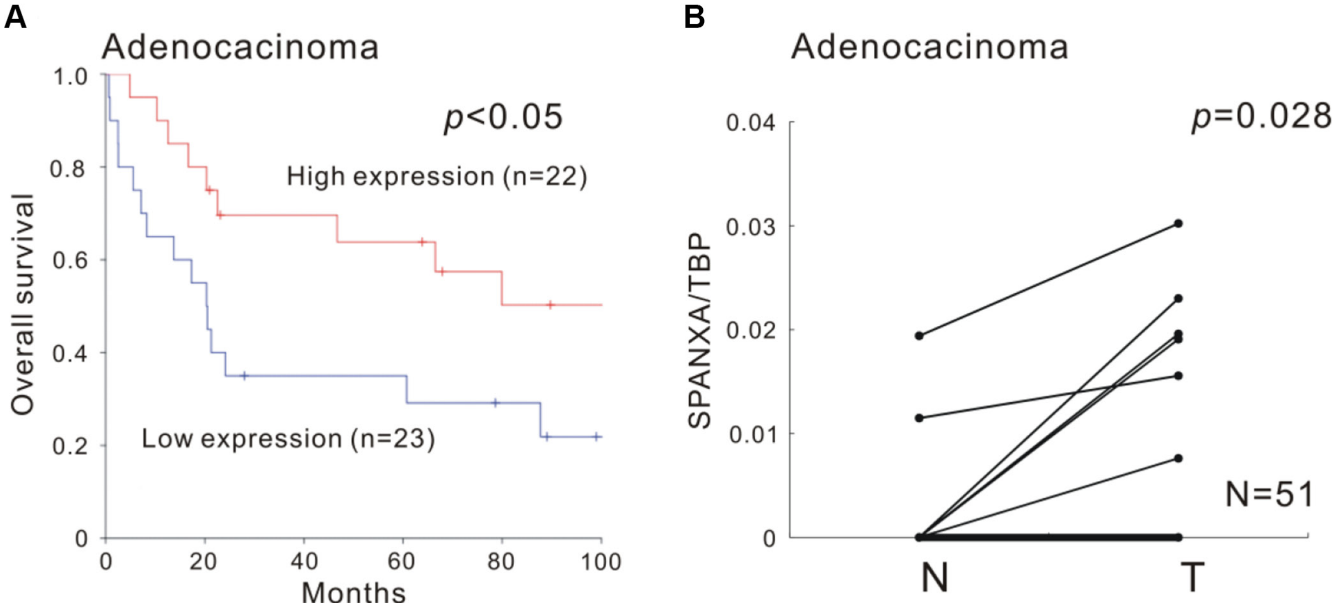

Supplementary Figure S2: SPANXA is associated with prolonged overall survival of patients with lung adenocarcinoma and expressed in tumor tissue. (**A**) The impact of SPANXA on the survival of 45 patients with lung adenocarcinoma was evaluated by Kaplan–Meier analysis. (**B**) Wilcoxon matched-pairs method was used to calculate the differential expression of SPANXA in the tumors and adjacent normal tissues.

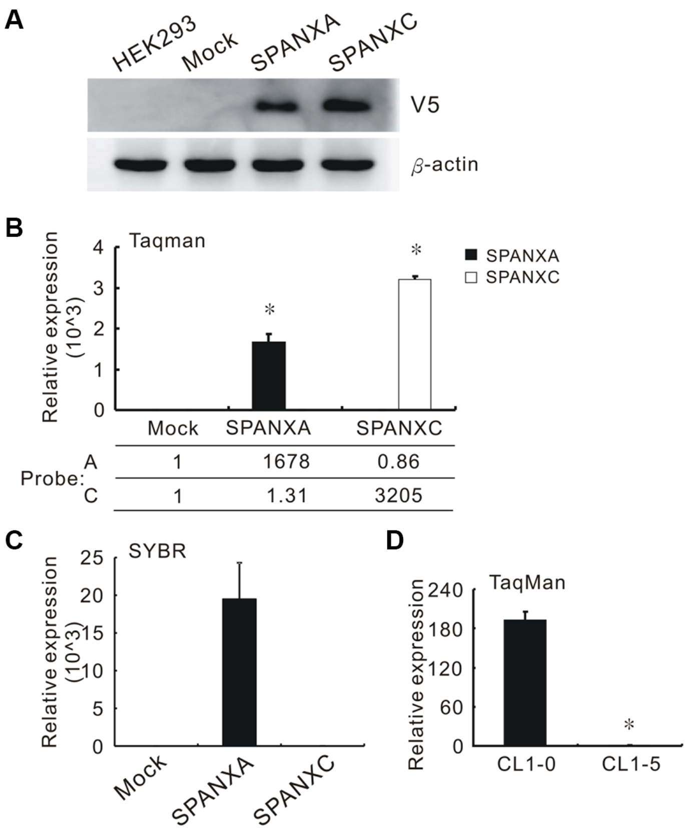

Supplementary Figure S3: Distinguishing SPANXA expression from SPANXC. (**A**) Ectopic expressions of SPANXA and SPANXC in HEK293 cells were assessed by immunoblot with anti-V5 antibody. (**B**) The specificity of customized SPANXA and SPANXC TaqMan probes was evaluated by TaqMan qRT-PCR for SPANXA- or SPANXC-expressing HEK293 cells. $*P < 0.05$ (mean ± SD, $n = 3$). (**C**) The specificity of customized SPANXA SYBR primers was evaluated by SYBR qRT-PCR. $*P < 0.05$ (mean ± SD, $n = 3$). (**D**) SPANXA expression in CL1-0 and CL1-5 cells was measured by TaqMan qRT-RCR. $*P < 0.05$ (mean ± SD, $n = 3$).

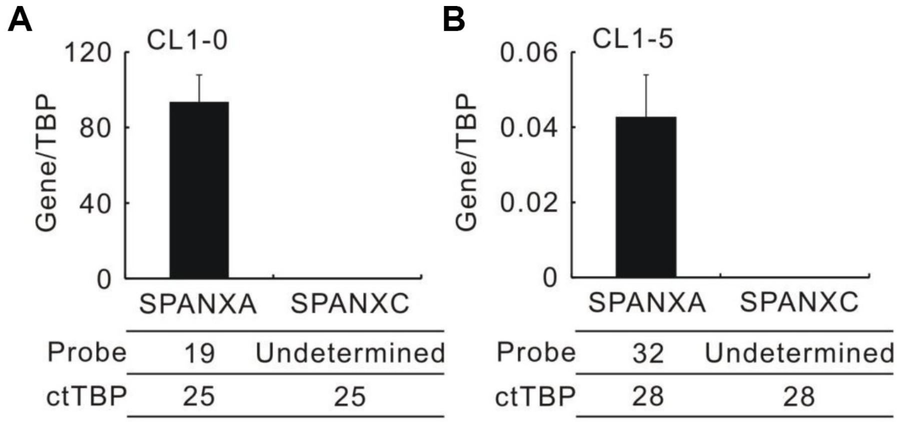

Supplementary Figure S4: The expression of SPANXC and SPANXA in CL1-0 and CL1-5. The expression of SPANXA and SPANXC are detected by TagMan probes in CL1-0 (**A**) as well as in CL1-5 (**B**). The exact cycle numbers are listed below.

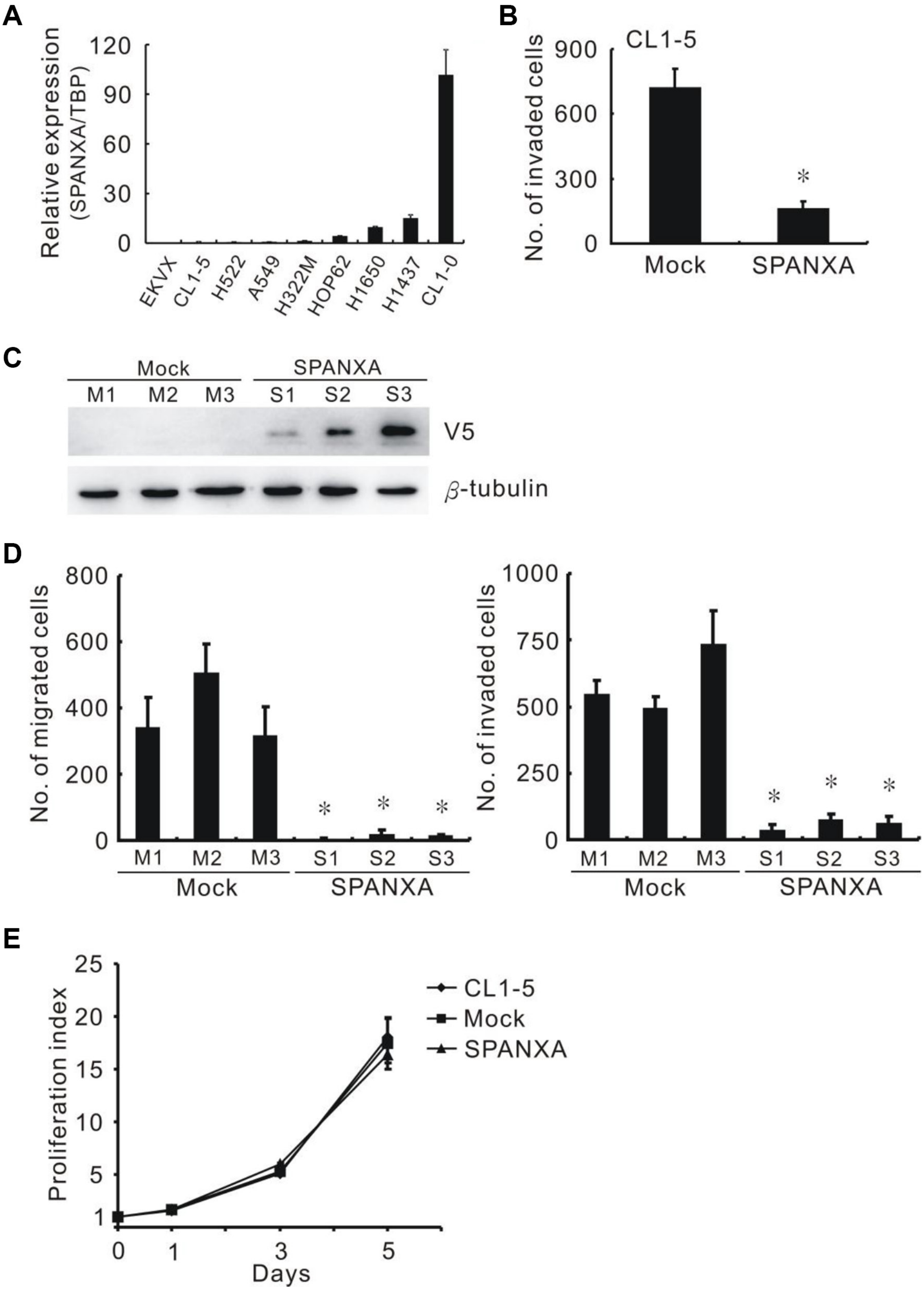

Supplementary Figure S5: SPANXA suppresses cell migration and invasion in transient and stable CL1-5 transfectants. (**A**) The *SPANXA* expression in different cell lines was measured by qRT-PCR. *$P < 0.05$ (mean ± SD, $n = 3$). (**B**) Invasion ability of mock or transiently SPANXA-expressing CL1-5 cells was measured by Transwell invasion assays. *$P < 0.05$ (mean ± SD, $n = 3$). (**C**) The ectopic expression of SPANXA in CL1-5 stably SPANXA-expressing single clones. (D) Cell migration and invasion abilities of stably SPANXA-expressing CL1-5 cells were measured by Transwell assays. *$P < 0.05$ (mean ± SD, $n = 3$). (**E**) MTT assay was performed for measuring the cell proliferation of the SPANXA-expressing CL1-5 cells. *$P < 0.05$ (mean ± SD, $n = 3$).

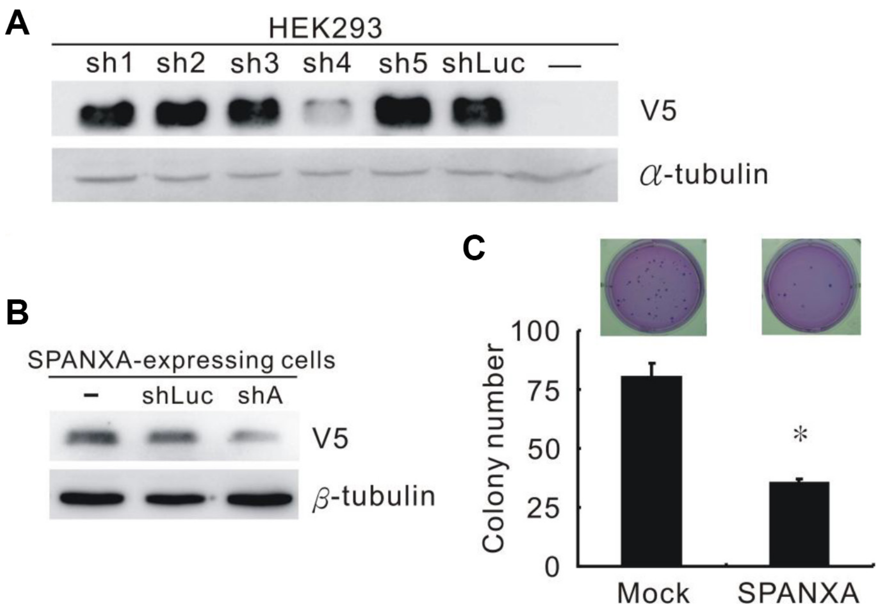

Supplementary Figure S6: Inhibition efficiency of shSPANXAs and the effect of SPANXA on anchorage-independent cell growth of CL1-5 cells. (**A**) Knockdown efficacy of different shSPANXAs was evaluated in SPANXA-expressing HEK293 cells. HEK293 cells were transiently transfected with the SPANXA-V5 expression vectors and followed by shSPANXA lentivirus infection. (**B**) Knockdown of SPANXA in SPANXA-expressing CL1-5 cells by shSPANXA lentivirus. (**C**) The impact of SPANXA on anchorage-independent cell growth. Colony formation ability of SPANXA-expressing CL1-5 was assessed by soft agar assays. *$P < 0.05$ (mean ± SD, $n = 3$).

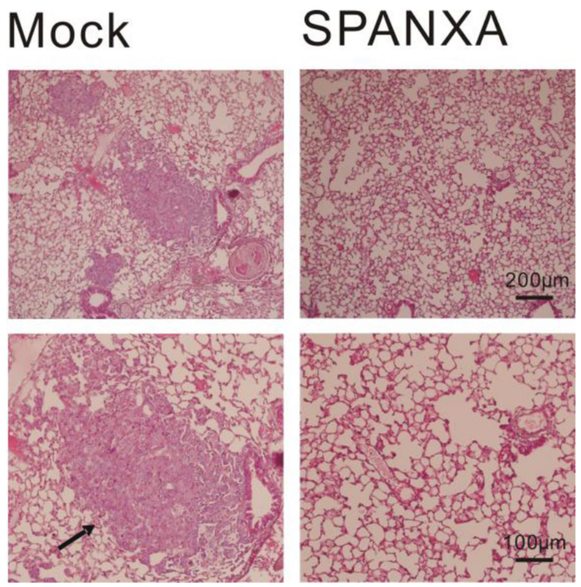

Supplementary Figure S7: Lung sections of metastatic tumor nudes. Metastatic lesions of lung sections were stained by hematoxylin and eosin (H&E) and indicated by the arrowhead. Scale bar in the upper panel, 200 μm. Scale bar in the lower panel, 100 μm.

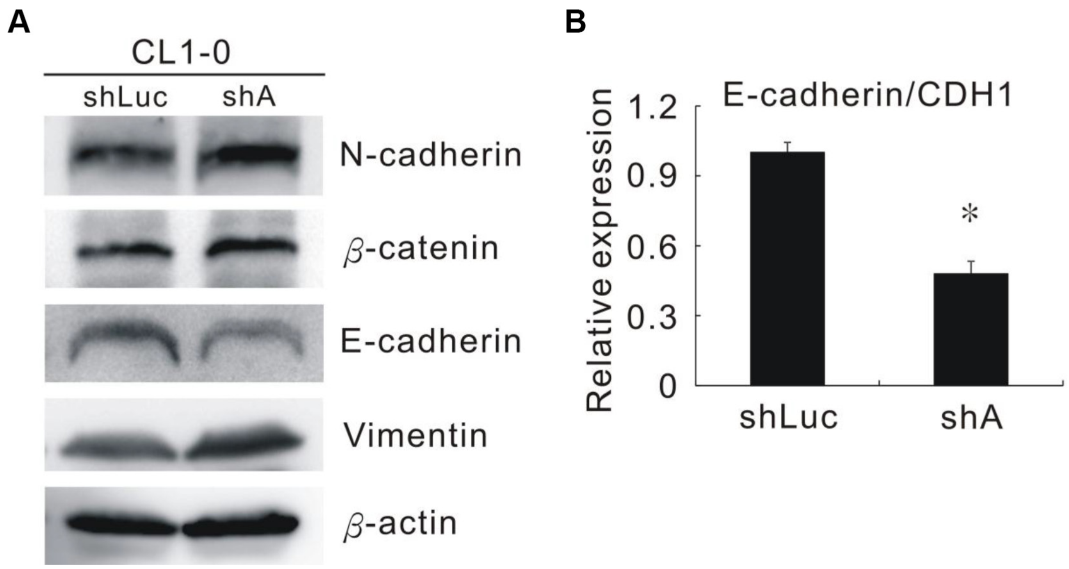

Supplementary Figure S8: The expressions of EMT markers are regulated by SPANXA. (**A**) The expression of EMT markers in SPANXA-silencing CL1-0 cells were measured by immunoblot assays. (**B**) The *E-caderin* expression in SPANXA-silencing CL1-0 cells was assessed by qRT-PCR. *$P < 0.05$ (mean ± SD, $n = 3$).

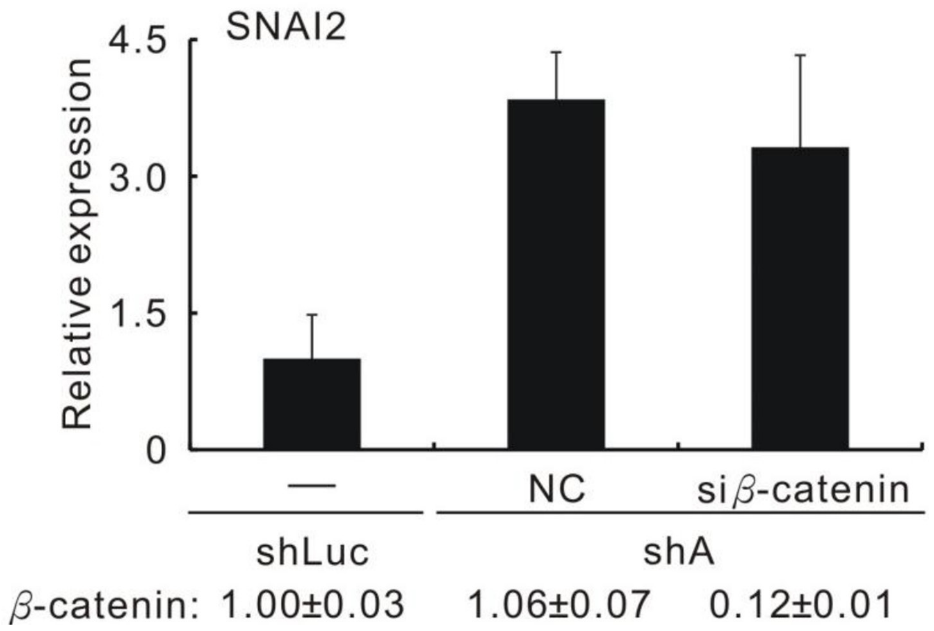

Supplementary Figure S9: Knockdown of β-catenin does not significantly reduce the SNAI2 expression in stably SPANXA-silencing CL1-0 cells. The lower row indicated the β-catenin relative expression (mean ± SD, $n = 3$).

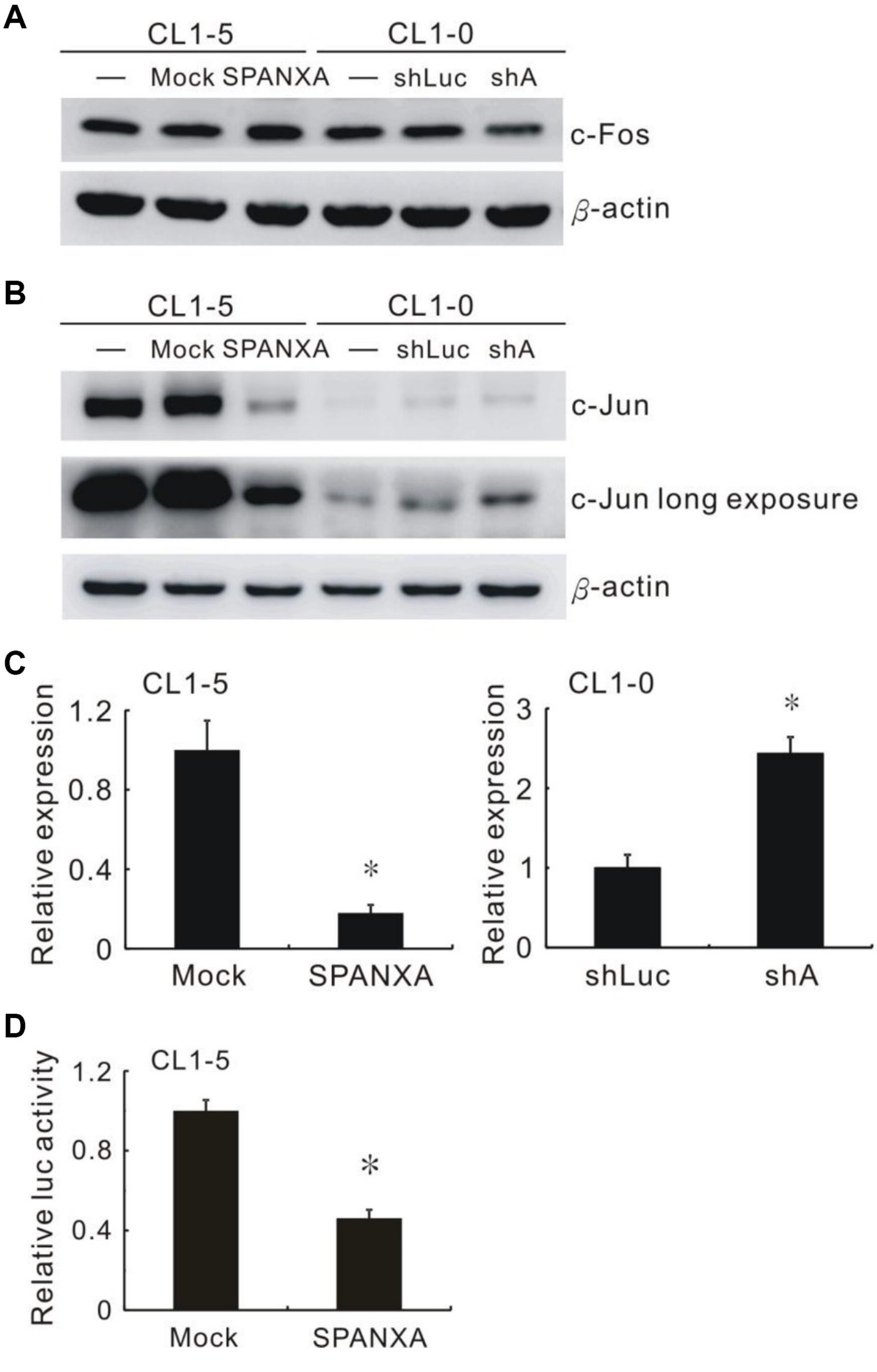

Supplementary Figure S10: c-FOS and c-JUN expression in CL pairs and SPANXA-expressing/silencing cells. (**A**) The c-FOS and (**B**) c-JUN expression in CL pairs and stably SPANXA-expressing/silencing cells were assessed by Western blotting. (**C**) c-JUN expressions in stably SPANXA-expressing CL1-5 cells and SPANXA-silencing CL1-0 cells were assayed by qRT-PCR. (**D**) AP-1 activity in stably SPANXA-expressing CL1-5 cells. *$P < 0.05$ (mean ± SD, $n = 3$).

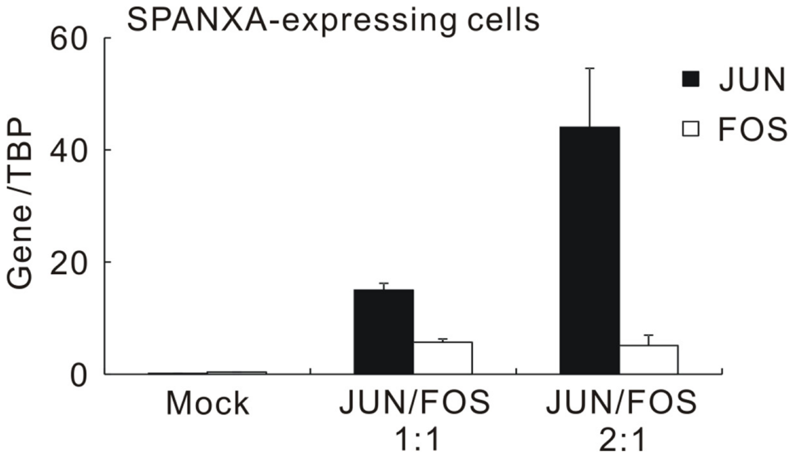

Supplementary Figure S11: Ectopic expressions of c-JUN and c-FOS in SPANXA-expressing cells. The different ratios of pcDNA3.1-JUN and pcDNA3.1-FOS plasmids were transfected into SPANXA-expressing CL1-5 cells, and the expressions of c-JUN and c-FOS were measured by qRT-PCR. *$P < 0.05$ (mean ± SD, $n = 3$).

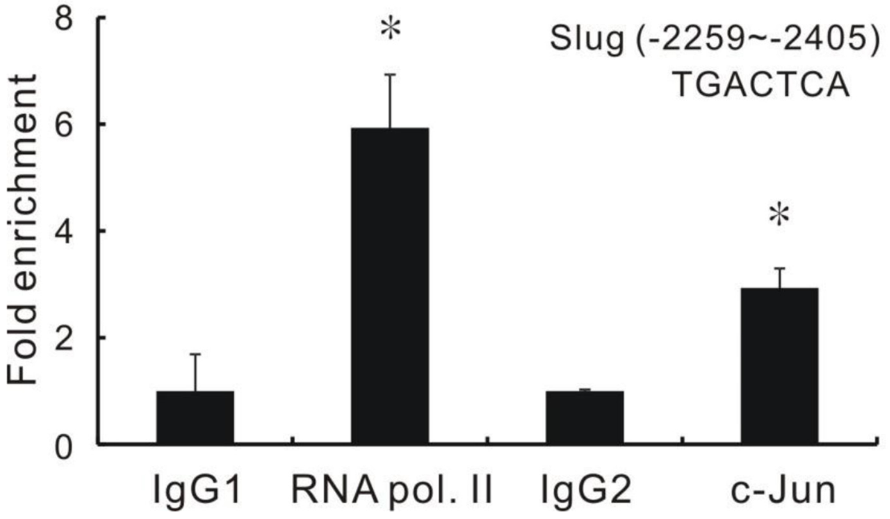

Supplementary Figure S12: Identification of AP-1 binding site in SNAI2 promoter. The c-JUN was ectopically expressed in HEK293 cells and then subjected to chromatin immunoprecipitation by 1ug anti-c-JUN antibody. The SNAI2 promoter was amplified by the specific primers. Anti-RNA polymerase II antibody served as the positive control. IgG1 and IgG2 serve as the negative control for anti-RNA polymerase II antibody and anti-c-JUN antibody, respectively. *$P < 0.05$ (mean ± SD, $n = 3$).

Supplementary Table S1: Clinical pathological characteristics of 97 lung cancer patients

	N (mean)	% (SD)
Gender		
Female	21	21.65
Male	76	78.35
Histology		
Adenocarcinoma	51	52.58
SCC	33	34.02
Others	13	13.40
Stage		
I	36	37.89
II	19	20.00
III	30	31.58
IV	10	10.53
Age		
Mean, SD	(68.47)	(10.48)
Hospital		
NTUH	32	32.99
TCVGH	65	67.01

Supplementary Table S2: The signal pathways altered by SPANXA

Top 10 pathways	*p*-value
*Regulation of epithelial-to-mesenchymal transition (EMT)	2.7e-8
#WNT signaling pathway	2.5e-7
*TGF-beta dependent induction of EMT via SMADs	4.6e-7
#ECM remodeling	1.9e-6
#TGF, WNT and cytoskeleton remodeling	1.8e-5
#Chemokines and adhesion	3.0e-5
Role of CDK5 in death and survival	4.4e-5
*TGF-beta dependent induction of EMT via RhoA, PI3K and ILK	4.5e-5
*TGF-beta dependent induction of EMT via MAPK	5.3e-5
#HGF signaling pathway	5.3e-5

*EMT pathways;
#EMT-related pathways.

Supplementary Table S3: The expression of SPANXA-altered genes and AP-1 targets

Gene*	Array (SPANXA/Mock)	qRT-PCR (SPANXA/Mock)	Function
CDH1	2.88	5.42 ± 0.79	
SNAI2	–4.43	–4.46 ± 0.15	
JUN	–3.03	**–5.57 ± 0.62**	
TGFBR2	–2.04	–1.84 ± 0.05	
MET	–2.01	–2.19 ± 0.30	
FN1	–2.28	–2.47 ± 0.14	EMT-related
VIM	–2.39	–3.04 ± 0.17	
HEY1	–3.34	–2.05 ± 0.04	
ETS1	–4.15	–5.71 ± 0.31	
TGM2	–2.96	–1.91 ± 0.22	
TWIST2	–2.32	–2.82 ± 0.13	
DUSP5	–2.30	–12.29 ± 1.27	
HMGB1	–2.07	ND	
ITGA6	–2.23	ND	
LAMC2	–2.08	ND	
MMP1	–2.38	ND	
NEFL	–2.06	ND	Metastasis
NRP1	–2.61	ND	
PLAU	–2.10	ND	
PLAUR	–2.35	ND	
RAC2	–2.31	ND	
S100A4	–3.03	ND	
IL6	–2.03	ND	
IL8	–5.03	ND	Chemoattraction
F3	–2.85	ND	
CXCL12	–2.54	ND	
ANGPT2	–2.58	ND	Angiogenesis
ABCA1	–4.79	ND	
GLRX	–4.69	ND	
ME1	–5.88	ND	
PDK1	–2.52	ND	Others
PSME3	–2.67	ND	
THBD	–3.16	ND	

ND: non-determined; *CDH1 is the only one of non-AP-1 targets.

Supplementary Table S4: GO process of the SPANXA-altered genes

Top 10 GO Processes	*p*-value
Regulation of cell motility	1.2e-22
Regulation of locomotion	6.4e-22
Regulation of cellular component movement	7.7e-22
Regulation of cell migration	4.2e-21
Cellular response to organic substance	8.0e-21
Positive regulation of cell motility	1.7e-20
Positive regulation of cellular component movement	2.7e-20
Positive regulation of locomotion	2.9e-20
Cellular response to chemical stimulus	6.7e-19
Positive regulation of cell migration	8.9e-19

Supplementary Table S5: AP-1 regulating targets enriched in SPANXA-altered genes

		SPANXA downregulated genes			
		Positive	Negative	Total	*p*-value*
AP-1 targets	Positive	31	552	583	1.50e-09
	Negative	366	20174	20540	
	Total	397	20726	21123	

*Chi-square test was used for the statistical analyses.

Supplementary Table S6: Primer list of SYBR qRT-PCR

Primer	Sequence (5′→3′)
DUSP-F	GGCTGACATTAGCTCCCACTTT
DUSP-R	CATGCAGATGGTGGGTGAAC
ETS1-F	CCTATCCAGAATCCCGCTATACC
ETS1-R	TGGAGCGTCTGATAGGACTCTGT
FN1-F	GAGCACCACCCCAGACATTACT
FN1-R	GACTCAGGTTATCAAAAGTGCAGGA
HEY1-F	AGAAGGCTGGTACCCAGTGCTTT
HEY1-R	AGTAACCTTTCCCTCCTGCCGT
JUN-F	GTCCACGGCCAACATGCT
JUN-R	CCGACGGTCTCTCTTCAAAATG
MET-F	AGCCAACCGAGAGACAAGCA
MET-R	TCCCACCACTGGCAAAGC
TGFBR2-F	CTGGGAGTTGCCATATCTGTCA
TGFBR2-R	AGTGCTCGCTGAACTCCATGA
TGM2-F	GACGATGGGTCTGTGCACAA
TGM2-R	GAGGACCCCTCTGGGTATTTG
TWIST2-F	CGCCAGGAGGAGATTCTGAA
TWIST2-R	CTCACTCCCGCCAACGTTT
CDH1-F	GAGCCTGAGTCCTGCAGTCC
CDH1-R	TGTATTGCTGCTTGGCCTCA
SNAI2-F	ATGCATATTCGGACCCACACATTA
SNAI2-R	AGATTTGACCTGTCTGCAAATGCT
VIM-F	TGAGTACCGGAGACAGGTGCA
VIM-R	TAGCAGCTTCAACGGCAAAGT
FOS-F<br>FOS-R	GGGCAAGGTGGAACAGTTATCTC<br>TCCGCTTGGAGTGTATCAGTCA
